# Perioperative morbidity of different operative approaches in early cervical carcinoma: a systematic review and meta-analysis comparing minimally invasive versus open radical hysterectomy

**DOI:** 10.1007/s00404-021-06248-8

**Published:** 2021-10-08

**Authors:** J. Kampers, E. Gerhardt, P. Sibbertsen, T. Flock, H. Hertel, R. Klapdor, M. Jentschke, P. Hillemanns

**Affiliations:** 1 Department of Gynecology and Obstetrics, Hannover Medical School, Carl-Neuberg-Str.1, 30625 Hannover, Germany; 2 Faculty of Economics and Management, Leibniz University Hannover, Hannover, Germany; 3 Comprehensive Cancer Center Niedersachsen (CCC-N), Hannover, Germany

**Keywords:** Early cervical cancer, Radical hysterectomy, Minimally-invasive, Laparoscopy, Robot-assisted, Postoperative morbidity

## Abstract

**Purpose:**

Radical hysterectomy and pelvic lymphadenectomy is the standard treatment for early cervical cancer. Studies have shown superior oncological outcome for open versus minimal invasive surgery, but peri- and postoperative complication rates were shown vice versa. This meta-analysis evaluates the peri- and postoperative morbidities and complications of robotic and laparoscopic radical hysterectomy compared to open surgery.

**Methods:**

Embase and Ovid-Medline databases were systematically searched in June 2020 for studies comparing robotic, laparoscopic and open radical hysterectomy. There was no limitation in publication year. Inclusion criteria were set analogue to the LACC trial. Subgroup analyses were performed regarding the operative technique, the study design and the date of publication for the endpoints intra- and postoperative morbidity, estimated blood loss, hospital stay and operation time.

**Results:**

27 studies fulfilled the inclusion criteria. Five prospective, randomized-control trials were included. Meta-analysis showed no significant difference between robotic radical hysterectomy (RH) and laparoscopic hysterectomy (LH) concerning intra- and perioperative complications. Operation time was longer in both RH (mean difference 44.79 min [95% CI 38.16; 51.42]), and LH (mean difference 20.96 min; [95% CI − 1.30; 43.22]) than in open hysterectomy (AH) but did not lead to a rise of intra- and postoperative complications. Intraoperative morbidity was lower in LH than in AH (RR 0.90 [0.80; 1.02]) as well as in RH compared to AH (0.54 [0.33; 0.88]). Intraoperative morbidity showed no difference between LH and RH (RR 1.29 [0.23; 7.29]). Postoperative morbidity was not different in any approach. Estimated blood loss was lower in both LH (mean difference − 114.34 [− 122.97; − 105.71]) and RH (mean difference − 287.14 [− 392.99; − 181.28]) compared to AH, respectively. Duration of hospital stay was shorter for LH (mean difference − 3.06 [− 3.28; − 2.83]) and RH (mean difference − 3.77 [− 5.10; − 2.44]) compared to AH.

**Conclusion:**

Minimally invasive radical hysterectomy appears to be associated with reduced intraoperative morbidity and blood loss and improved reconvalescence after surgery. Besides oncological and surgical factors these results should be considered when counseling patients for radical hysterectomy and underscore the need for new randomized trials.

## Introduction

Surgical therapy of early cervical cancer (FIGO Stadium ≤ IIA) is primarily recommended by national and international guidelines [1]. Miscellaneous surgical approaches were established to perform radical hysterectomy and lymphadenectomy. According to mostly retrospective studies abdominal radical hysterectomy appears to be associated with a higher rate of morbidities such as bladder dysfunction, longer hospital stay or postoperative infection compared to laparoscopic radical hysterectomy (LH) [2–4].

Systematic reviews showed the superiority of laparoscopic hysterectomy regarding inoperative blood loss, hospital stay and postoperative complications over the abdominal approach [5–7].

In addition, these reviews reported similar oncological outcomes between LH and AH which led to the wide implementation of LH as a standard approach in early cervical cancer [6].

The publication of the LACC (Laparoscopic Approach to Cervical Cancer) trial in 2018, the first large multicenter randomized controlled trial comparing AH with LH approaches in early cervical cancer, led to a drastic change of recommendations for operative treatment [8]. The LACC trial showed a reduced overall- (OS) and disease-free survival (DFS) in the minimally invasive therapy group, compared to open radical hysterectomy. A reduction of perioperative morbidity in the minimally invasive therapy group was not shown in this prospective trial, either.

These results contradicted previous results of systematic reviews comparing laparoscopic and robotic (RH) with open surgical approaches [6]. Robot-assisted operations, which have been introduced into the gynecologic oncologic operative field a decade ago [9], were included in the minimally invasive results in this study. In previous meta-analyses [10–12] a non-inferiority of robot-assisted approaches regarding perioperative complication rates compared to LH or AH was shown.

This meta-analysis was performed to evaluate the morbidities and clinical outcomes of cervical cancer patients treated by RH, LH or AH.

## Materials and methods

The methods for this study were specified a priori based on the recommendations in the Preferred Reporting Items for Systematic Reviews and Meta-Analyses (PRISMA) statement [13].

### Search strategy

A systematic database research for studies comparing RH with LH or AH for the treatment of early cervical cancer via Ovid-Medline and EMBASE without restriction of publication year was performed. Search terms combined MESH-terms (uterine neoplasms) or Emtree headings and the related terms “cervical cancer”, “uterine cancer”, “cervical neoplasm”, as well as “laparoscopic surgery”, “hysterectomy”, “Wertheim operation”, “Robotics,” and “robotic-assisted surgery”.

### Study selection

**Fig. 1:**
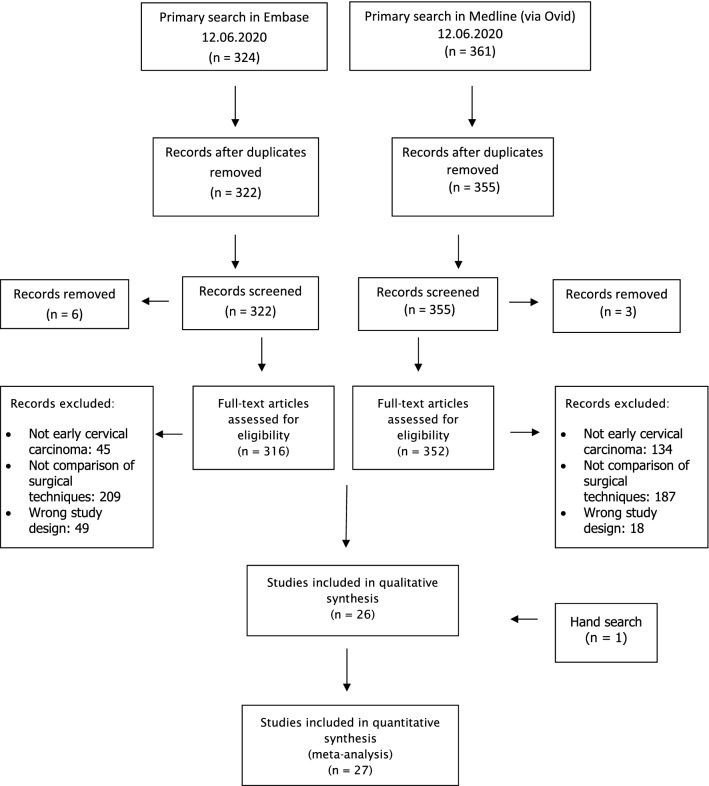
PRISMA flowchart

Study selection was done independently by JK and EG. In case of conflicting opinions PH decided about inclusion or exclusion. The inclusion criteria were adapted to the inclusion criteria of the LACC trial [8] and specified 1. studies that included patients with early cervical cancer FIGO IA1, IA2, IB1, IB2, IIA1, 2. comparative studies between RH or OH or LH, 3. studies that reported at least one outcome of interest, and 4. published original, peer-reviewed articles. Only studies with complete publication of all results were considered. Non-original studies, animal or preclinical trials, abstract-only publications, reports in a language other than English or German and duplicates were excluded. All reasons for exclusion are mentioned in the Preferred Reporting Items for Systematic Reviews and Meta‐Analyses flowchart (Fig. 1). One study already presented at ESGO congress prior to the systematic research was added by hand search upon publication.

The algorithms used for primary search as well as the full list of search results can be found in the supplementary items. If possible, the authors of studies that were only published as congress abstracts were tried to be contacted via email and asked to provide their data.

### Data extraction and quality assessment

**Fig. 2:**
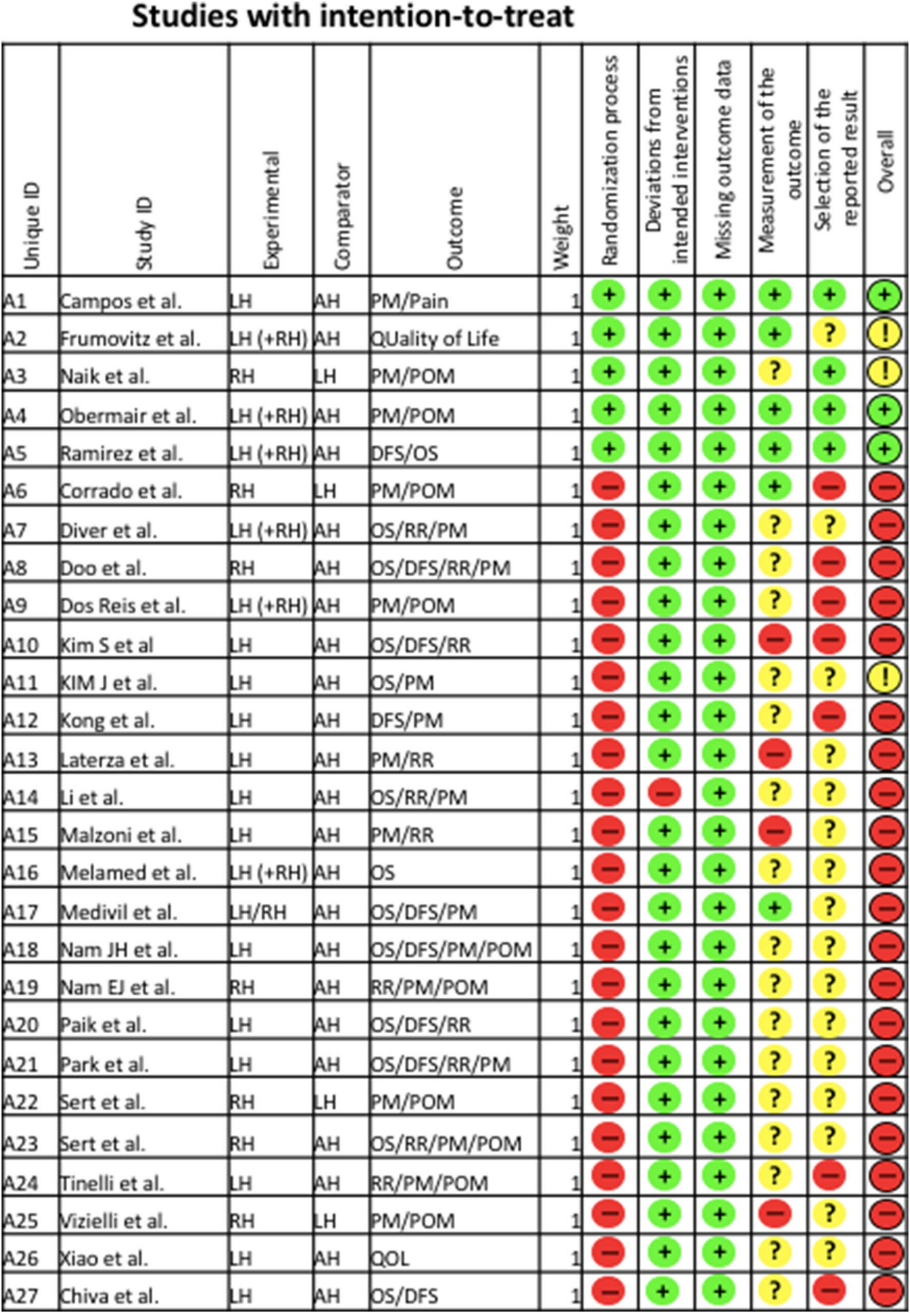
RoB

The updated Cochrane risk of bias tool 2 (RoB 2) was used to assess the scientific quality of the included studies [14] (Fig. 2). The quality assessments were performed by two independent researchers (JK and EG). Disagreements were resolved by consensus of all authors.

Two reviewers independently extracted the safety and effectiveness indexes into a pre-specified data extraction form and double checked them.

### Statistical analysis

Inter-study heterogeneity was assessed using the maximum likelihood estimator with calculation of *τ*2 and its corresponding *p* value [15]. This *p* value indicates the probability that deviation from inter-study homogeneity can be explained by chance with a lower *p* value implying significant heterogeneity. The 95% confidence intervals (CI) were used as the summary variables for continuous outcomes and the risk rate (RR) and 95% CI for dichotomous variables.

Statistical analysis was conducted by fixed-effect models in the absence of significant heterogeneity and random-effect models in the presence of significant heterogeneity. Analysis was by intention to treat. Subgroup analyses were prespecified according to the study design and the year of publication (before or after the LACC study data publication).

## Results

685 studies met the inclusion criteria and were assessed for eligibility. After removing records with no full text, wrong study designs (e.g. reviews) wrong patient selection (e.g. chemotherapy before operation) and duplicates, 27 suitable comparative studies were included into final analysis. Table 1 shows the characteristics of the 27 studies.

**Table 1:** Study characteristics: AH abdominal hysterectomy LH laparoscopic hysterectomy na not available RCT randomized controlled study RH robotic hysterectomy

Author	Region	Publication year	Study year	FIGO Stage	Study design	Cohort	LH	RH	AH	Median Follow-up (months) LH/AH
Campos et al. [25]	Brazil	2013	1999–2004	IA2-IB	RCT, single center	LH:AH	16	0	14	na
Bogani et al. [26]	Worldwide	2020	2013–2014	IB1	Retrospective, multicenter	LH(+ RH):AH	291	na	402	59/56
Corrado et al. [27]	Italy	2014	2010–2012	IA1-IIA1	Retrospective, multicenter	LH:RH	30	30	0	25
Diver et al. [28]	USA	2016	2000–2013	IA1-IIB	Retrospective, multicenter	LH(+ RH):AH	101 (71)	na	282	61.2
Doo et al. [29]	USA	2019	2010–2016	IB1	Retrospective, multicenter	RH:AH	0	49	56	na
Dos Reis et al. [30]	USA	2018	1990–2013	IA1, IA2, IB1, IIA1	Retrospective, single center	LH(+ RH):AH	121 (50)	na	427	10.4
Frumovitz et al. [31]	Worldwide	2020	2008–2017	IA1, IA2, IB1	RCT, multicenter	LH(+ RH):AH	319 (45)	na	312	36
Kim S et al. [32]	Korea	2019	2000–2018	IB1-IB2	Retrospective, multicenter	LH:AH	343	0	222	59.1
Kim J et al. [33]	Korea	2018	2011–2014	na	Retrospective, multicenter	LH:AH	3100	0	3235	na
Kong et al. [34]	Korea	2014	2006–2013	IB-IIA	Retrospective, single center	LH:AH	40	0	48	28/58
Laterza et al. [35]	Italy	2016	1997–2014	IA1, IA2, IB1, IIA1	Retrospective, single center	LH:AH	82	0	68	121.2/43.5
Li et al. [36]	China	2007	1998–2005	IB-IIA	Retrospective, single center	LH:AH	90	0	35	26
Malzoni et al. [37]	Italy	2009	1995–2007	IA1, IA2, IB1	Retrospective, single center	LH:AH	65	0	62	52.5/71.5
Melamed et al. [38]	USA	2018	2000–2013	IA2, IB1	Retrospective, single center	LH(+ RH):AH	1225 (978)	na	1236	45
Mendivil et al. [39]	USA	2015	2009–2013	IA2-IIB	Retrospective, single center	LH:RH:AH	49	58	39	39
Naik et al. [40]	UK	2010	2005–2007	IB1	RCT, single center	LH:AH	8	0	7	na
Nam JH et al. [41]	Korea	2011	1997–2008	IA2-IIA	Retrospective	LH:AH	263	0	263	92
Nam EJ et al. [42]	Korea	2010	2006–2009	IA2-IIB	Retrospective, single center	RH:AH	0	32	32	15.3
Obermair et al. [43]	Worldwide	2020	2008–2017	IA1, IA2, IB1	RCT, multicenter	LH(+ RH):AH	279 (41)	na	257	6
Paik et al. [44]	Korea	2019	2000–2008	IB-IIA	Retrospective, multicenter	LH:AH	119	0	357	63.6
Park et al. [45]	Korea	2016	1997–2013	IA2-IIA	Retrospective, single center	LH:AH	186	0	107	58.8
Ramirez et al. [46]	Worldwide	2018	2008–2017	IA1, IA2, IB1	RCT, multicenter	LH(+ RH):AH	319 (45)	na	312	30
Sert et al. [47]	Norway	2007	2004–2005	IA1-IB1	Retrospective, single center	LH:RH	7	7	0	14/25
Sert et al. [48]	Norway	2016	2005–2011	IA1-IB2	Retrospective, multicenter	RH:AH	0	259	232	34.6/35.2
Tinelli et al. [49]	Italy	2011	2003–2010	IA1-IIA	Retrospective, multicenter	LH:RH	76	23	0	46.5/24.5
Vizzielli et al. [50]	Italy	2016	2013–2015	IA2-IIB	Retrospective, multicenter	LH:RH	42	21	0	na
Xiao et al. [51]	China	2016	2001–2014	IA-IIA	Retrospective, single center	LH:AH	42	0	16	46.1/51.2

The studies were performed in the USA, Asia, Europe and Brazil. The publication years ranged from 2007–2020. 15.713 patients with operative treatment of early cervical carcinoma were included. 8.021 patients were treated with open surgery and 479 by robotic surgery, respectively. 7.213 patients received laparoscopic surgery. Five prospective, randomized-control trials were included. The design of 22 studies design was retrospective. One study [16] compared all three operative approaches. 19 studies compared LH to AH, of which seven compared LH including RH to AH. Four studies compared LH to RH and three RH to AH.

The statistical assessment revealed a large overall risk of bias since most of the included studies were neither randomized nor prospective. All included studies were assessed regarding potential conflicts of interest. In all studies the ICMJE uniform disclosure form was completed.

Subgroups were created concerning the endpoints examined (estimated blood loss (EBL), mean hospital stay (HS), operation time (OT), intraoperative morbidity (IM), postoperative morbidity (POM), the study design (randomized controlled trials (RCT)/retrospective), and the date of publication (before/after the LACC trial). Survival rates including overall and disease-free survival were published by our research group in 2021 [17].

### Estimated blood loss (EBL)

**Fig. 3:**
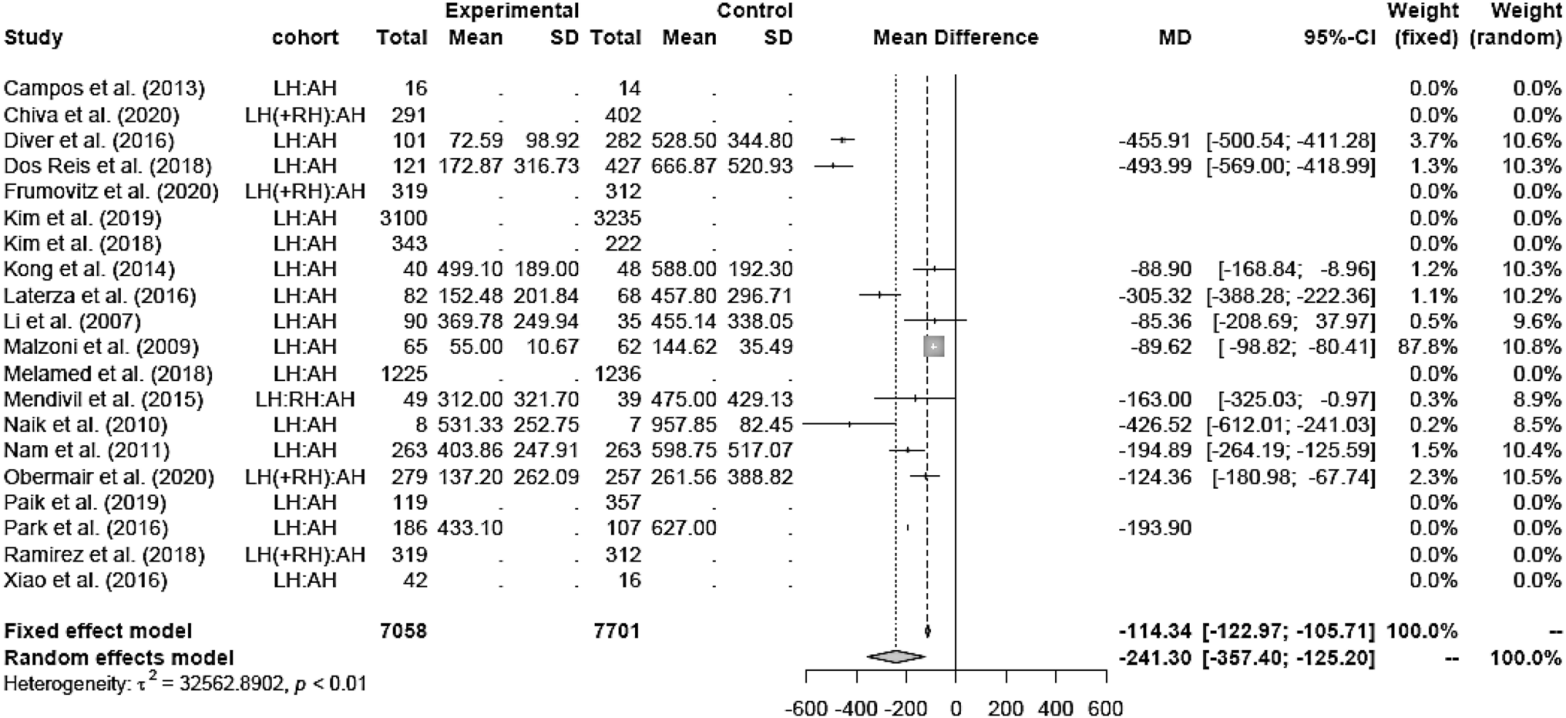
Estimated blood loss LH versus AH

**Fig. 4:**
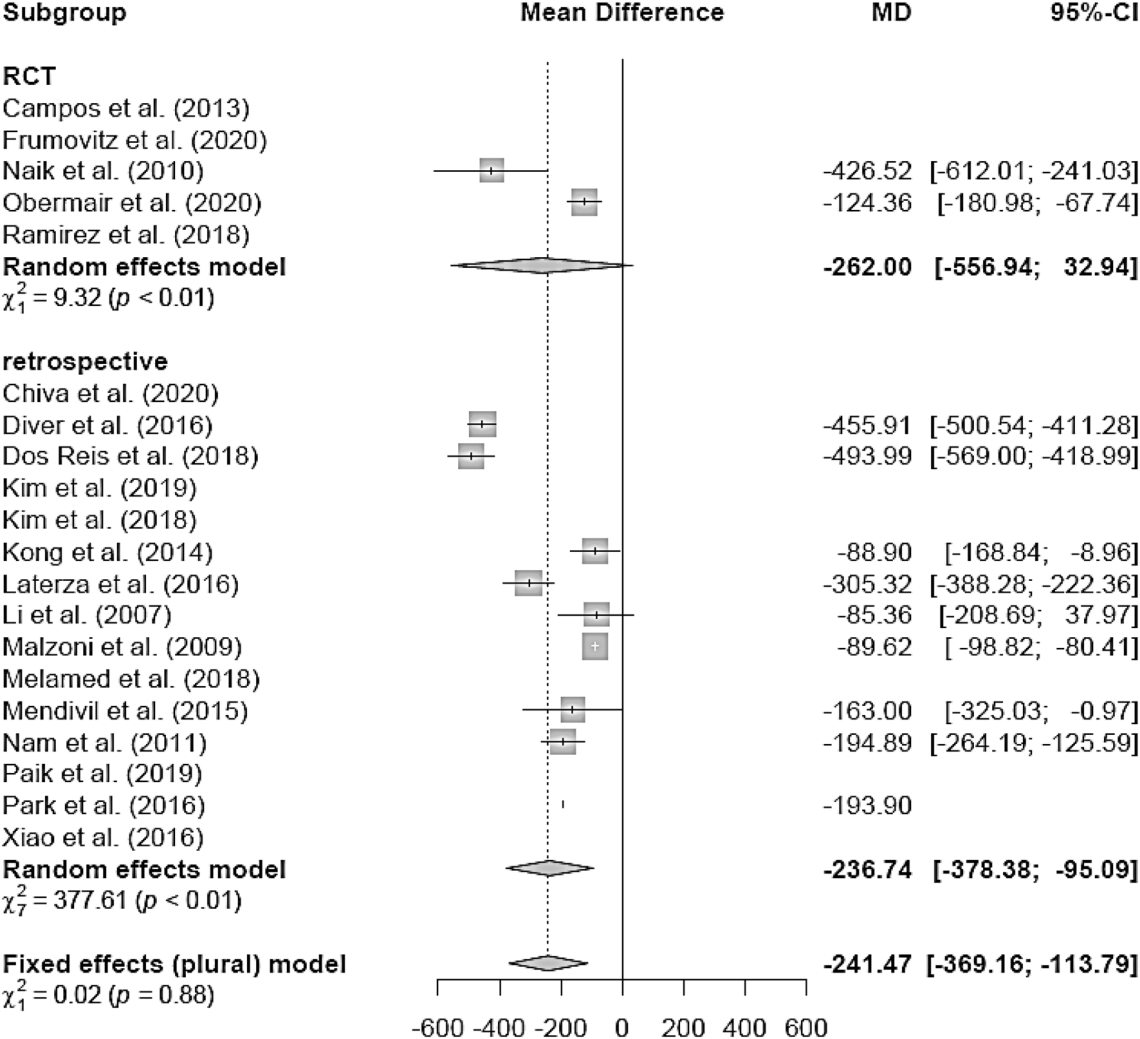
Estimated blood loss LH vs AH (study design)

**Fig. 5:**
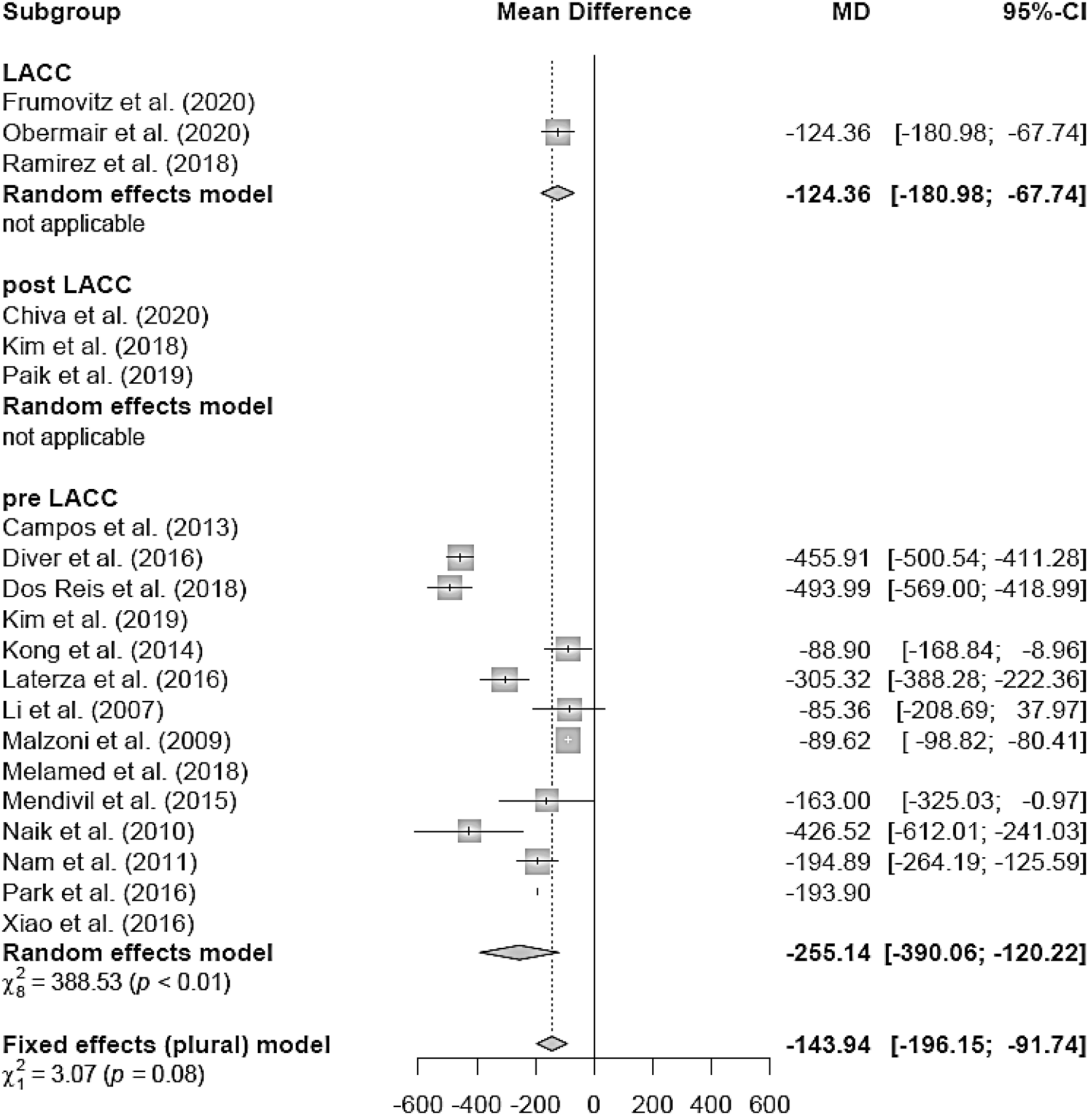
Estimated blood loss LH vs AH (date of publication)

The EBL (in milliliters) was lower in the LH group (mean 311.5 ml) than in the AH group (mean 462.27 ml; mean difference (MD) − 114.34 [− 122.97; − 105.71]) (Fig. 3). The LH group of retrospective studies presented with lower EBL in the LH group (MD − 241.47 [− 369.16; − 113.79]), whereas the RCT (MD − 262.00 [− 555.94; 32.94]) showed no difference between the two groups (Fig. 4). There were no post-LACC studies but only pre-LACC studies that showed a lower blood loss for LH (Fig. 5).

**Fig. 6:**
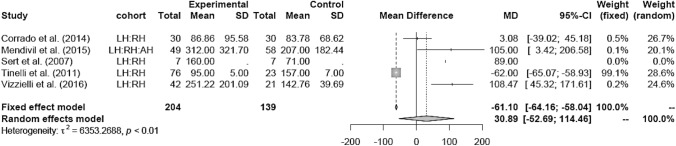
Estimated blood loss LH vs RH

LH versus RH (mean 140.98 ml) showed mixed results. Due to high heterogeneity, the random effects model was applied and revealed no significant difference between the two groups (MD 30.89 [− 52.69; 114.46]) (Fig. 6).

**Fig. 7:**
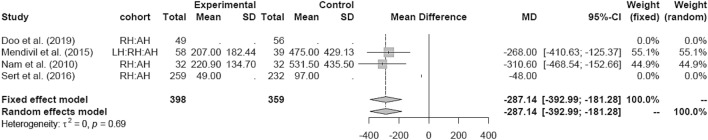
Estimated blood loss RH vs AH

The blood loss in RH was significantly lower than in AH (MD − 287.14 [− 392.99; − 181.28]) (Fig. 7).

Subgroups of study design and date of publications in the LH versus RH and RH versus AH groups did not provide different results due to the lack of studies in either one of the groups.

### Mean hospital stay (HS)

**Fig. 8:**
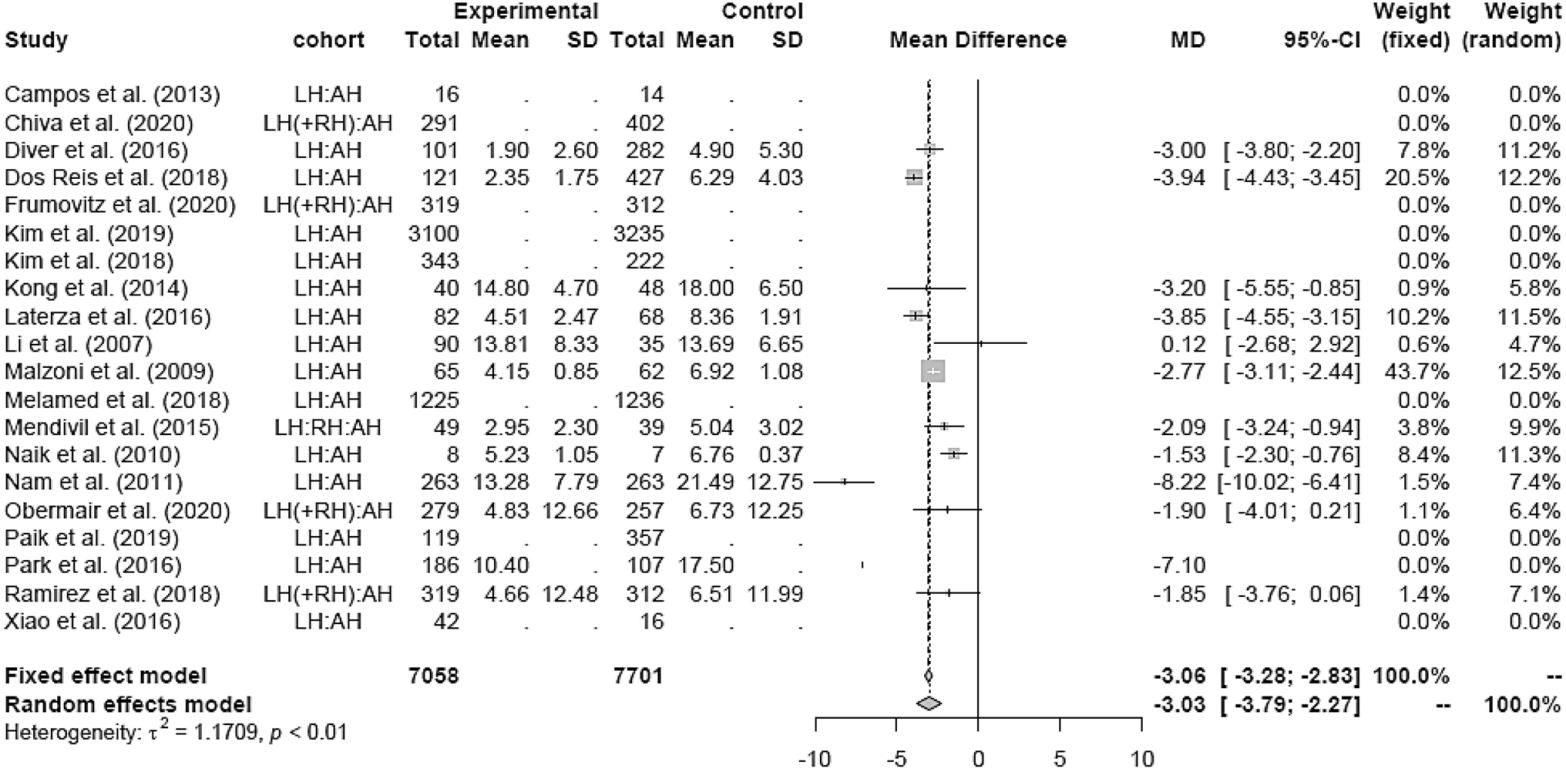
Hospital stay LH vs AH

**Fig. 9:**
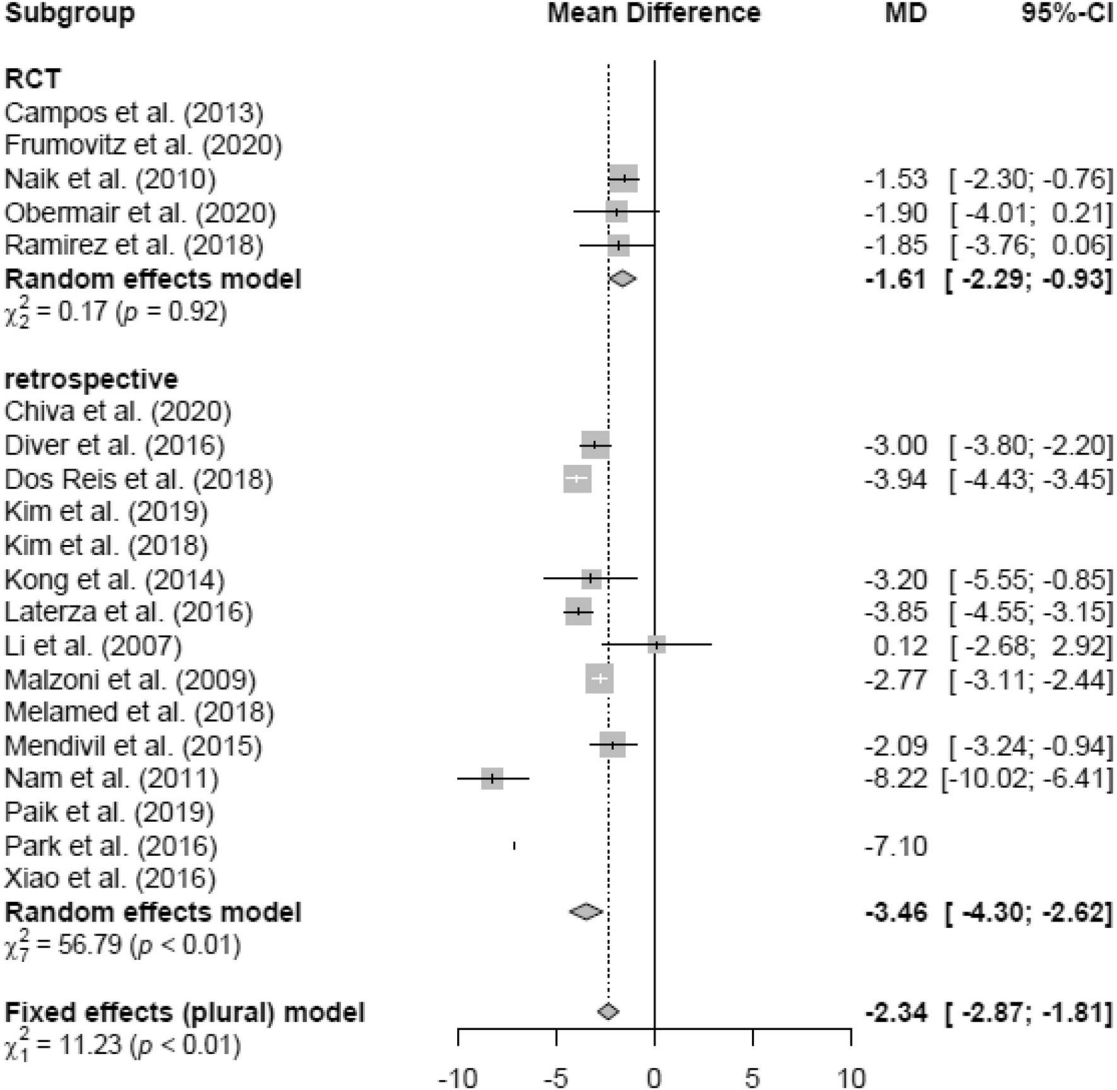
Hospital stay LH vs AH (study design)

**Fig. 10:**
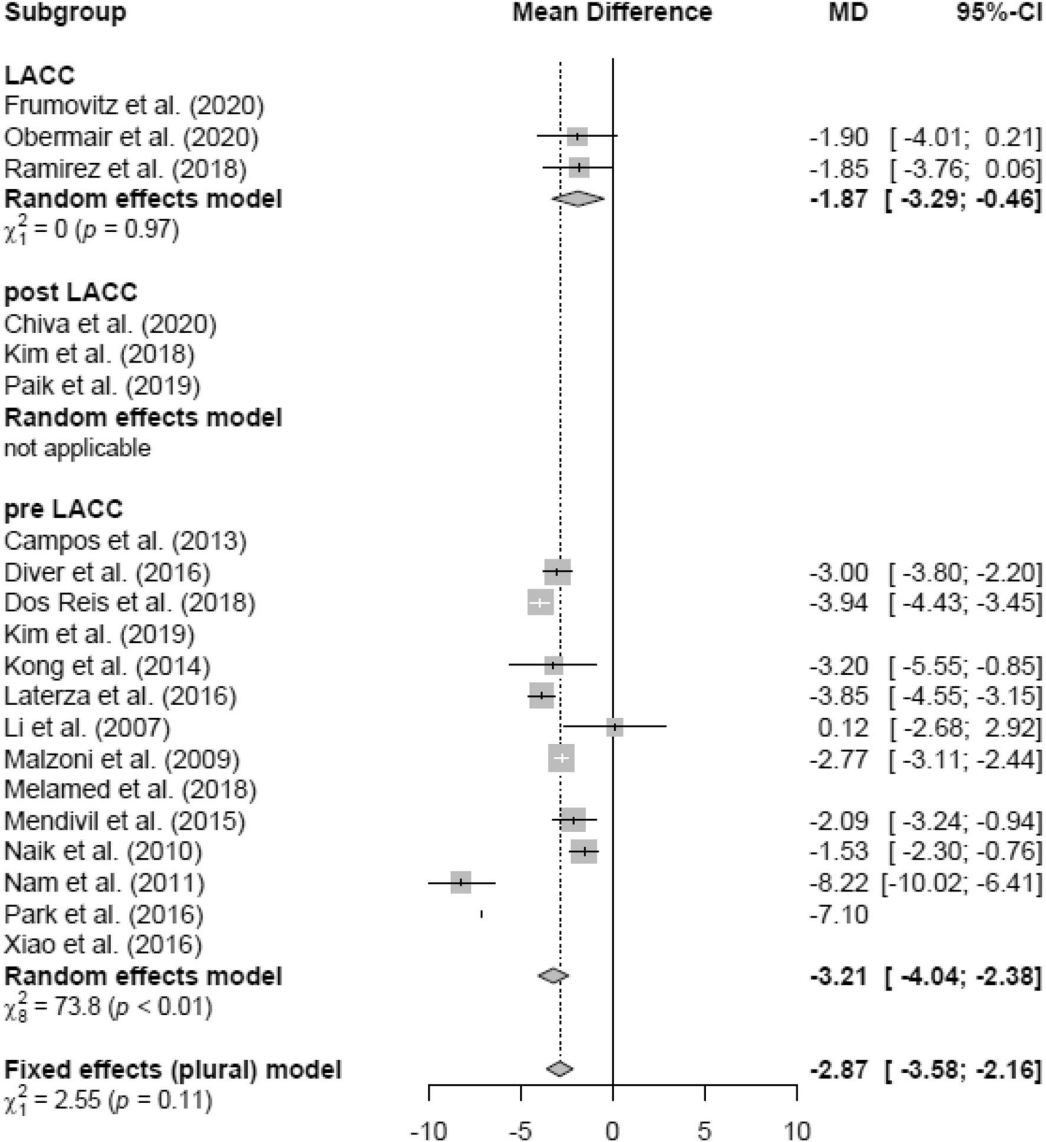
Hospital stay LH vs AH (date of publication)

The duration of HS (in days) of patients in the LH group (mean 7.98 days) was significantly lower than in the AH group (mean 12.02 days; MD − 3.06 [− 3.28; − 2.83]) (Fig. 8). In the RCT and retrospective and pre-LACC groups, HS was significantly lower in the LH group than in the AH group, respectively (Figs. 9, 10). None of the post-LACC studies, however, examined the HS.

**Fig. 11:**
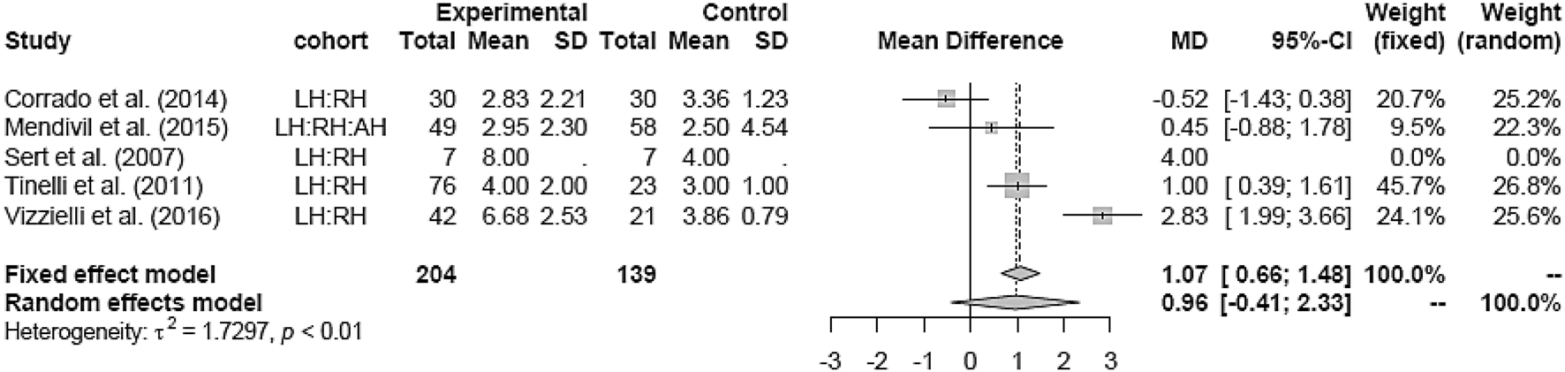
Hospital stay LH vs RH

The LH versus RH groups (mean 4.58 days) showed mixed results. Due to high heterogeneity the random-effects model was used and showed no significant difference in HS (MD 1.07 [0.66; 1.48]) (Fig. 11).

**Fig. 12:**
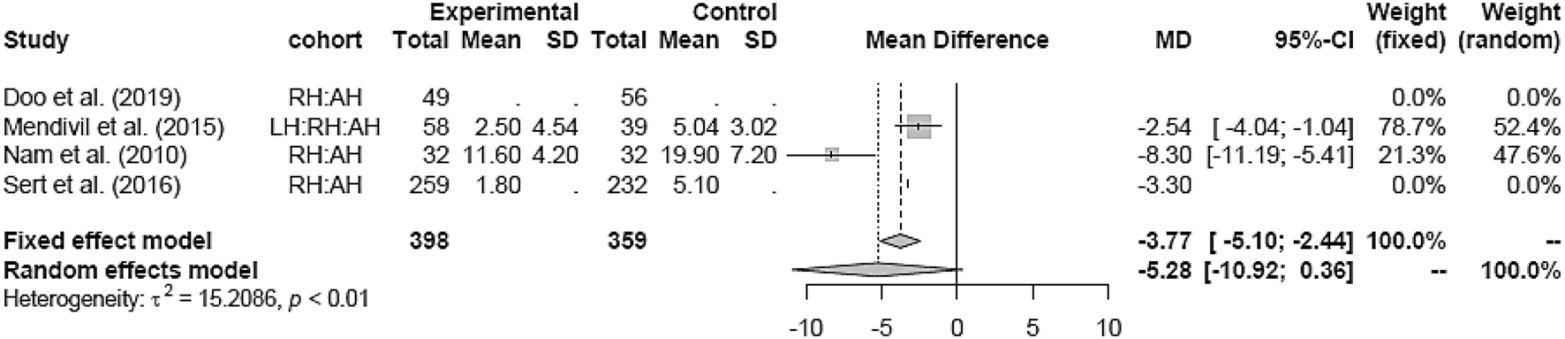
Hospital stay RH vs AH

In comparison to AH, RH was associated with a shorter hospital stay (MD − 3.77 [− 5.10; − 2.44]) (Fig. 12).

Subgroup analysis for study design and date of publications in the LH versus RH and RH versus AH groups could not be assessed due to the lack of studies in either one of the groups.

### Operation time (OT)

**Fig. 13:**
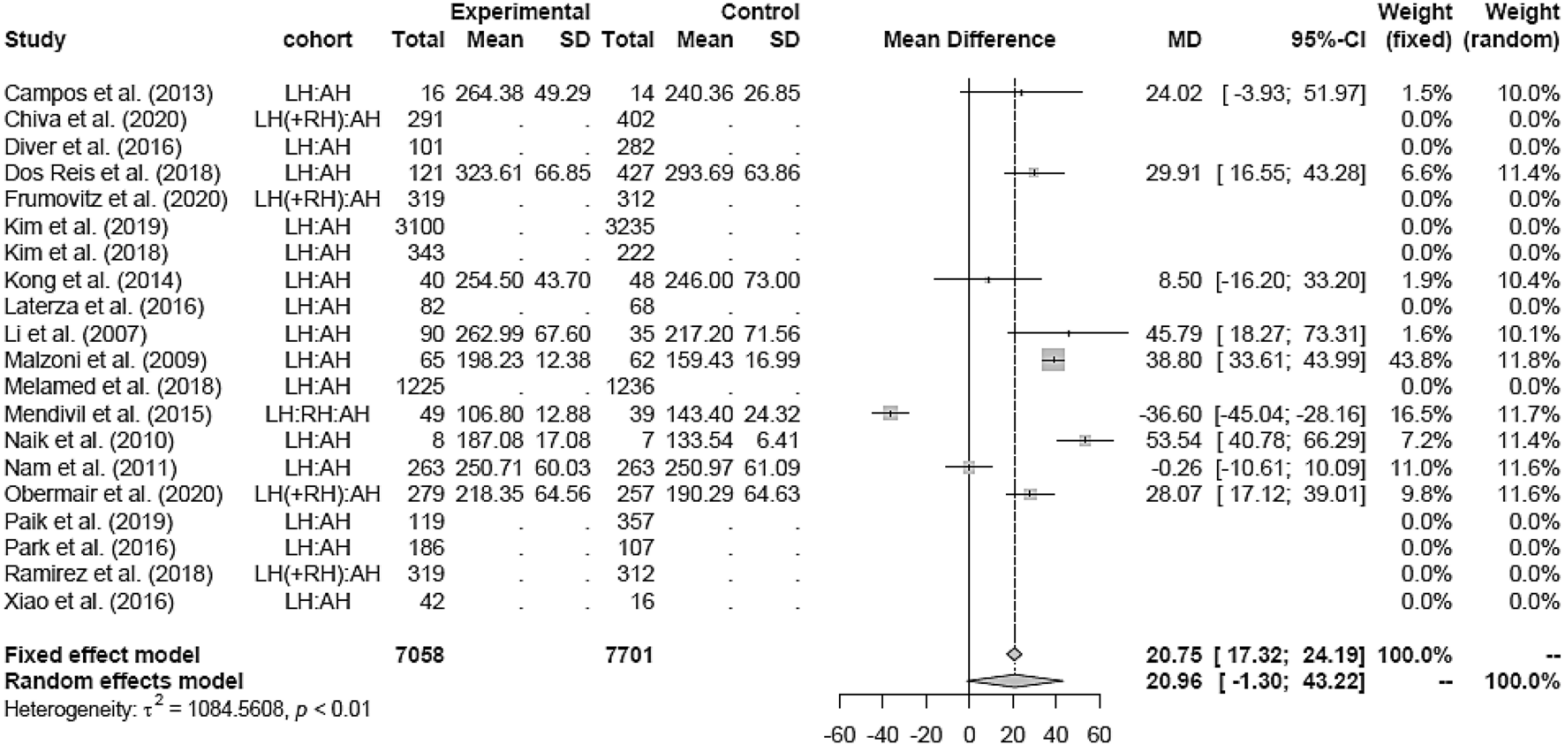
Operation time LH vs AH

**Fig. 14:**
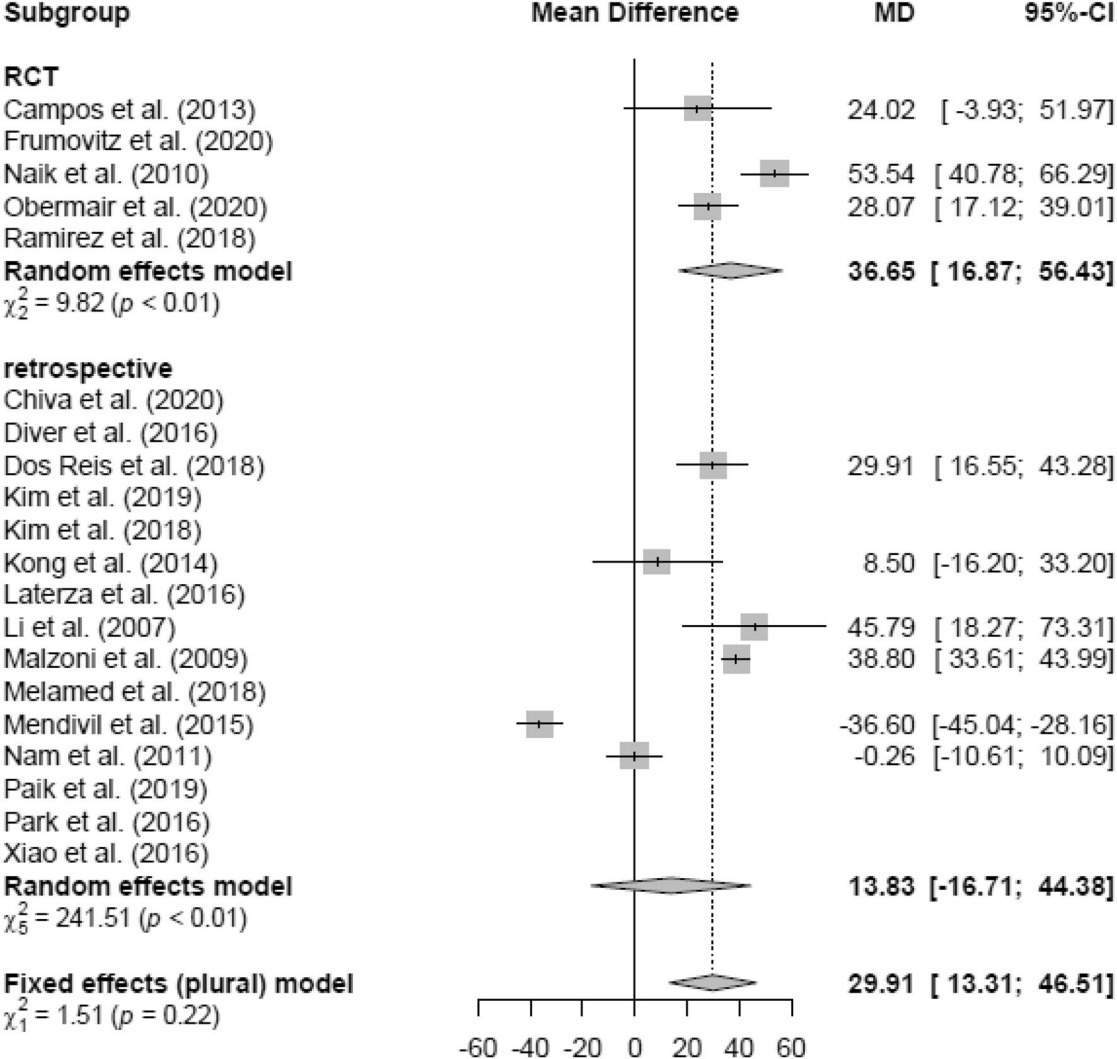
Operation time LH vs AH (study design)

**Fig. 15:**
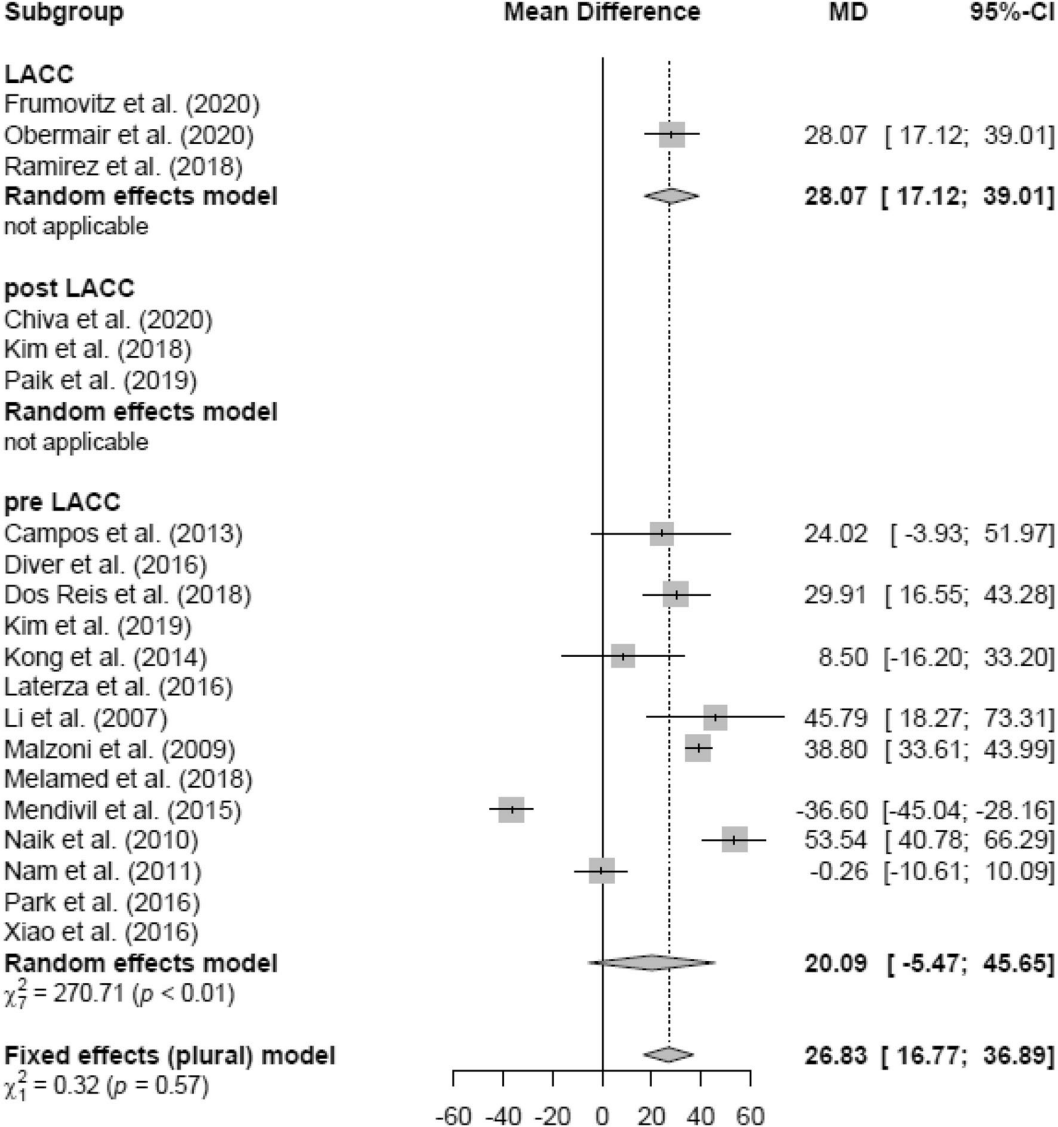
Operation time LH vs AH (date of publication)

Due to high heterogeneity, the random effects model was used in the LH versus AH analysis. LH (mean 267.37 min) showed a non-significantly longer OT (in minutes) than AH (mean 213.89 min; MD 20.96 [− 1.30; 43.22]) (Fig. 13). Similar results were found in the RCT and retrospective subgroups, respectively (Figs. 14, 15).

**Fig. 16:**
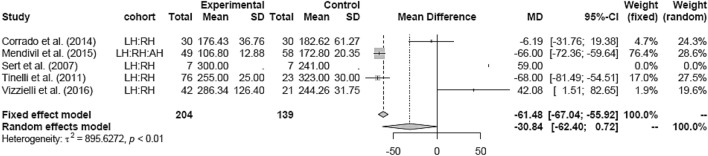
Operation time LH vs RH

Random effects analysis was also used comparing LH and RH (mean 250.70 min). The result showed no difference in OT between both groups (Fig. 16).

**Fig. 17:**
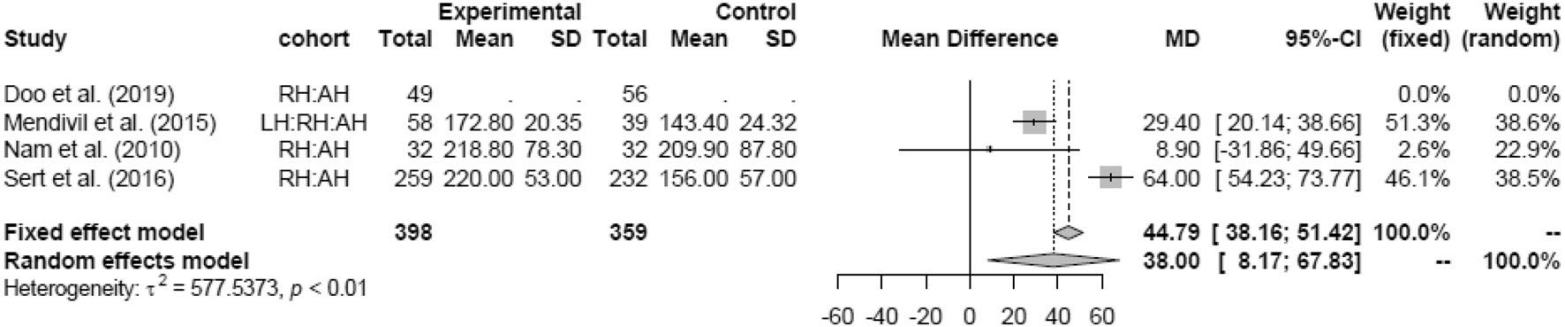
Operation time RH vs AH

OT was significantly longer in RH than in AH (MD 44.79 [38.16; 51.42]) (Fig. 17).

Subgroup analysis for study design and date of publications in the LH versus RH and RH versus AH groups could not be assessed due to the lack of studies in either one of the groups.

### Intraoperative morbidity (IM)

**Fig. 18:**
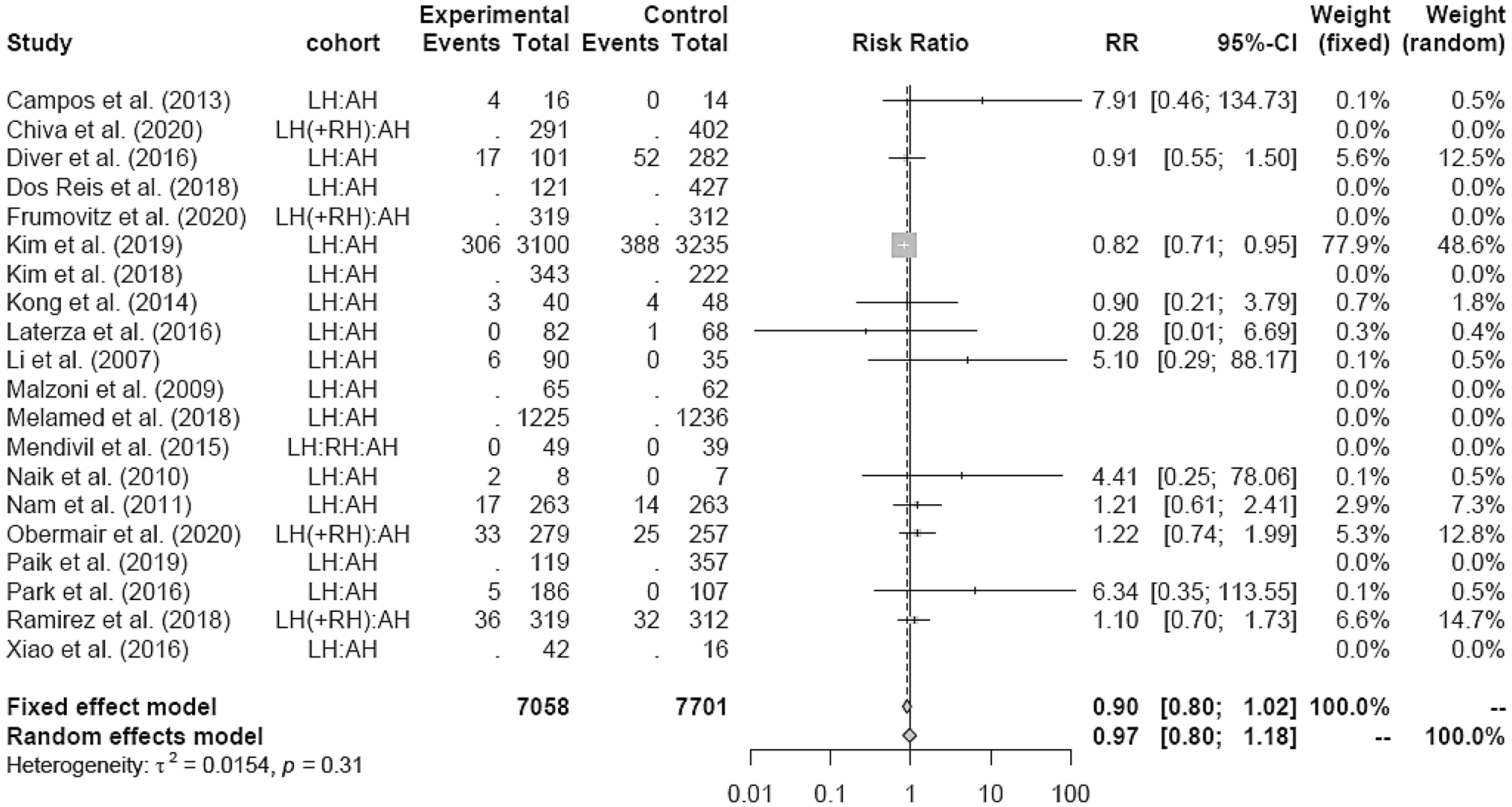
Intraoperative morbidity LH vs AH

**Fig. 19:**
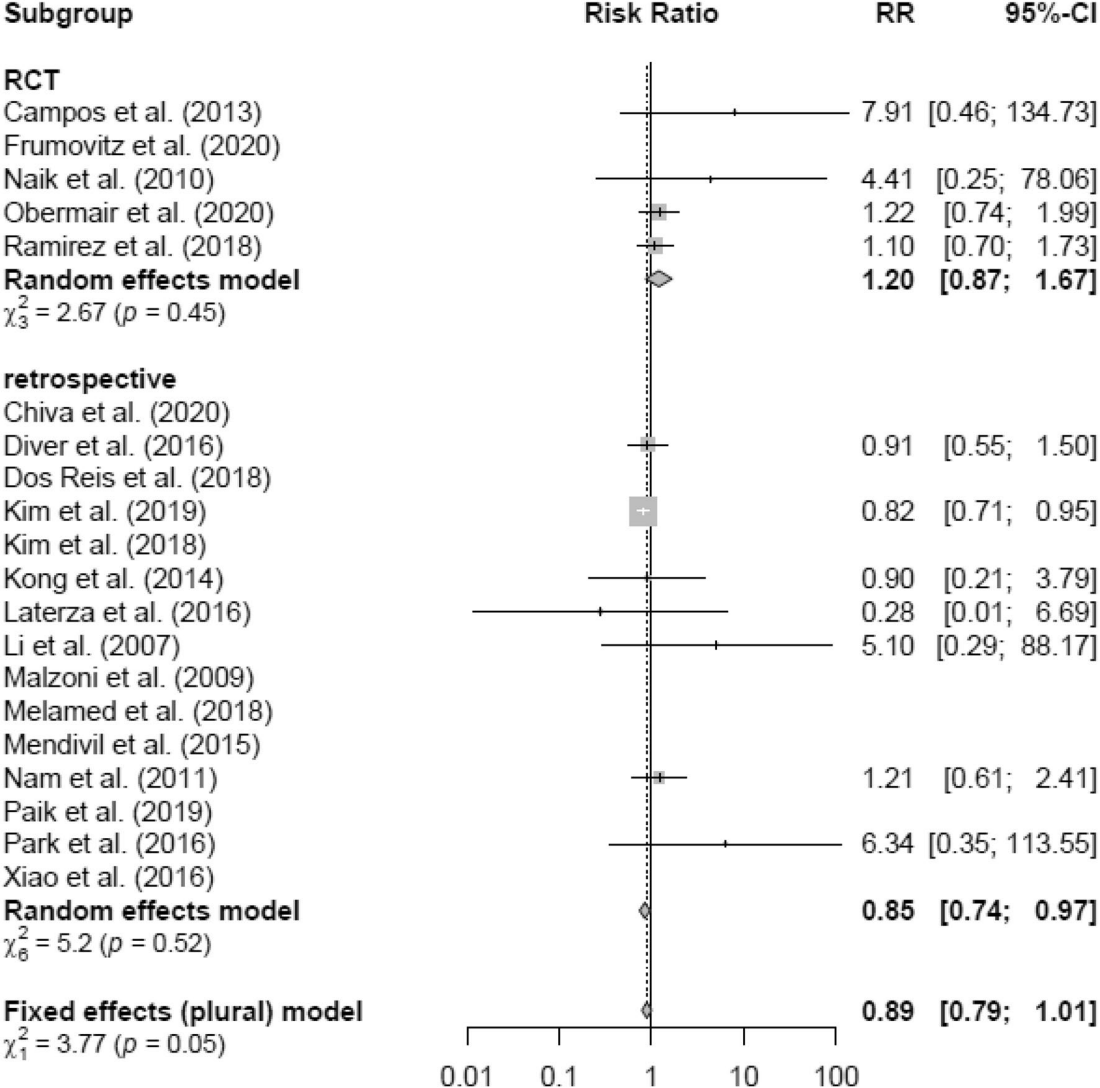
Intraoperative morbidity LH vs AH (study design)

Intraoperative morbidity included vascular, bladder, urethral and nerve injury, as well as transformation to open surgery. There was no difference in IM in LH versus AH studies (RR 0.90 95% CI [0.80; 1.02]) (Fig. 18) overall and in the RCT group, respectively. The retrospective subgroup showed a lower IM in LH (RR 0.85; [0.74; 0.97]) (Fig. 19). There were no post LACC studies evaluating IM.

**Fig. 20:**
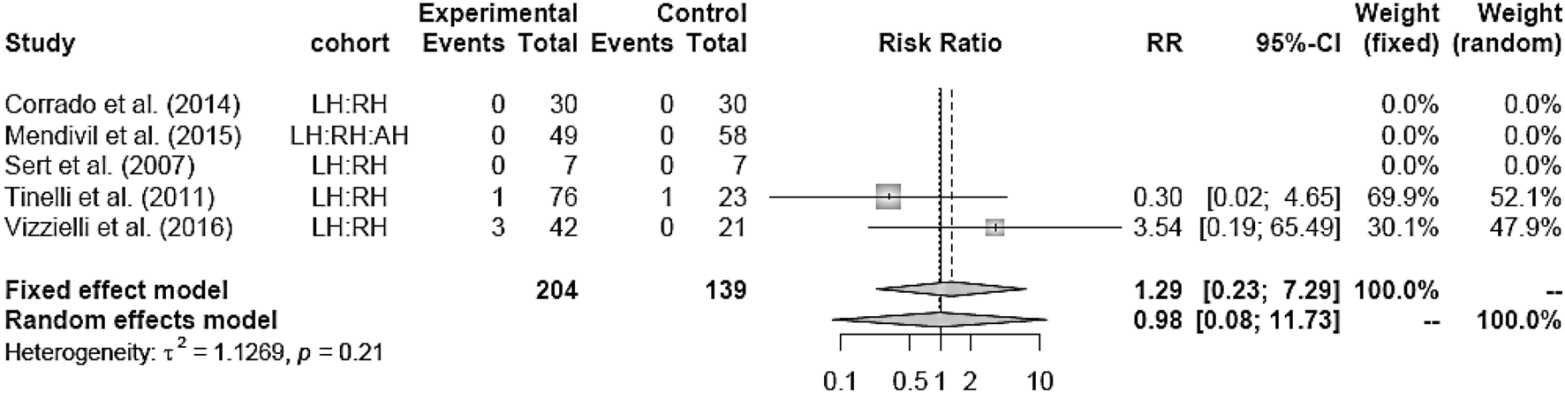
Intraoperative morbidity LH vs RH

**Fig. 21:**
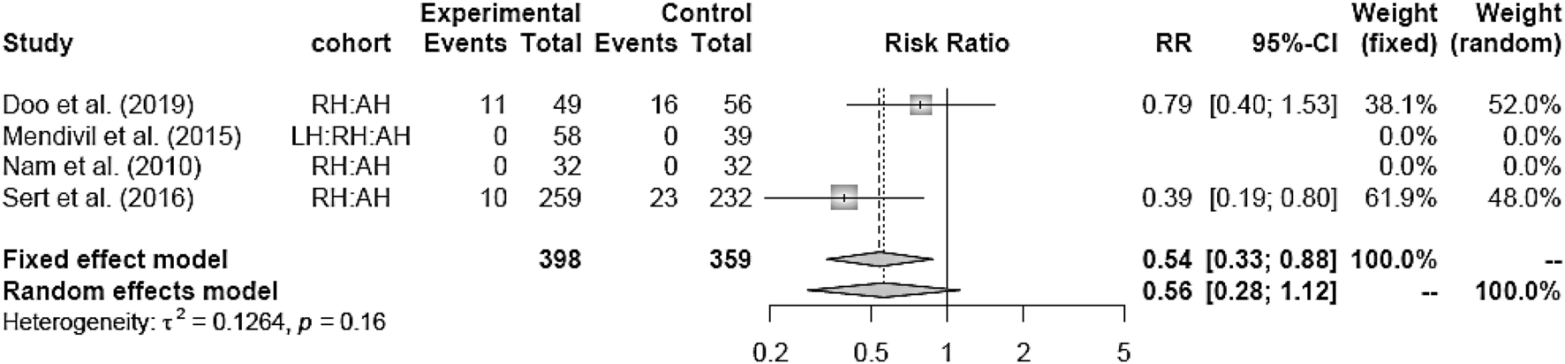
Intraoperative morbidity RH vs AH

LH versus RH showed no difference as well (Fig. 20). RH showed lower IM compared to AH (RR 0.54 [0.33; 0.88]) (Fig. 21).

Subgroup analysis for study design and date of publications in the LH versus RH and RH versus AH group could not be assessed due to the lack of studies in either one of the groups.

### Postoperative morbidity (PM)

**Fig. 22:**
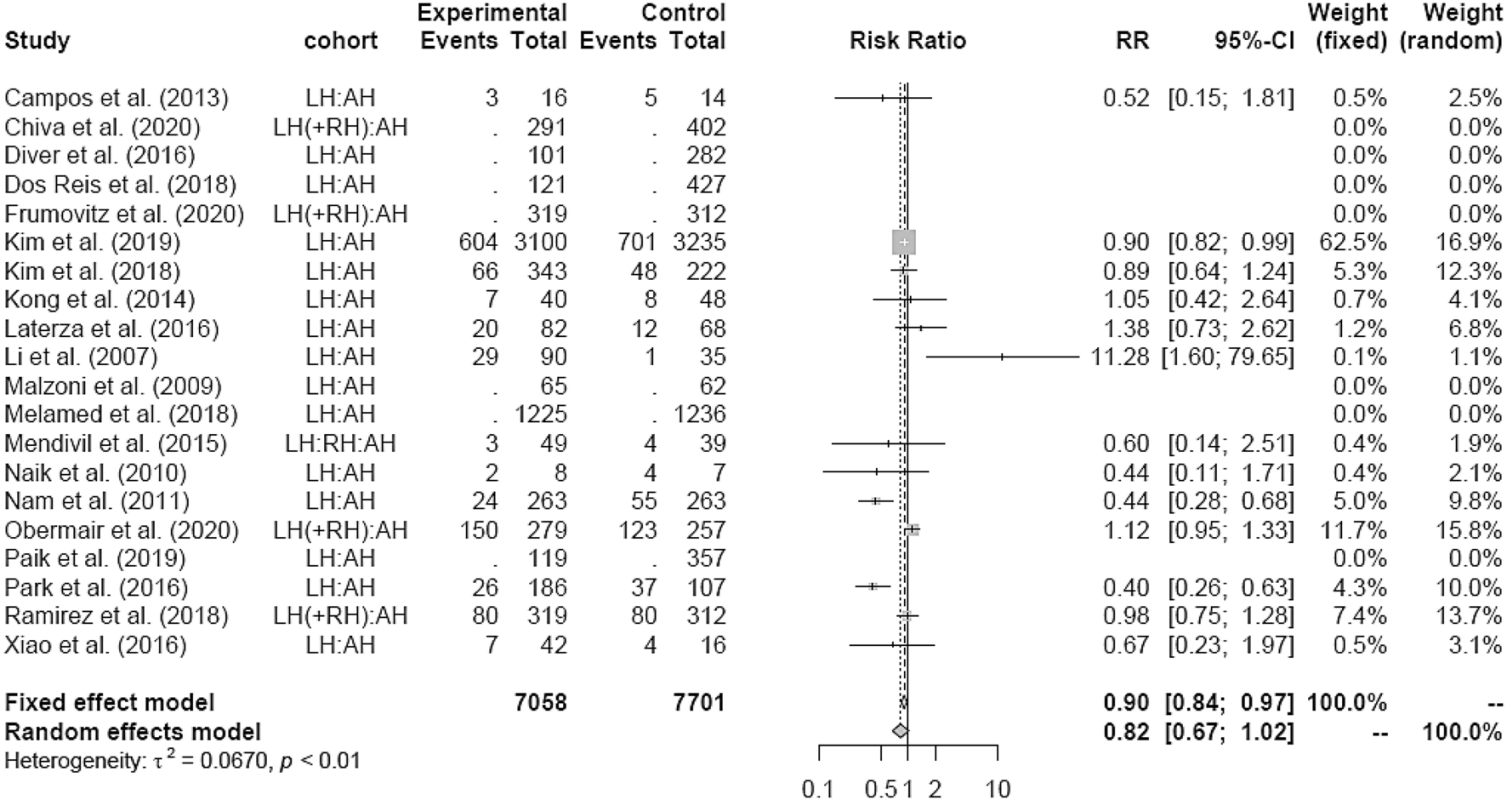
Postoperative morbidity LH vs AH

Postoperative morbidity included infections, abscess formation, urinary dysfunctions and incontinence as well as thrombosis and fistula. Due to high heterogeneity, the random effects model was used in the LH versus AH analysis and showed no significant difference (Fig. 22). RCT and retrospective design as well as the date of publication led to no different result.

**Fig. 23:**
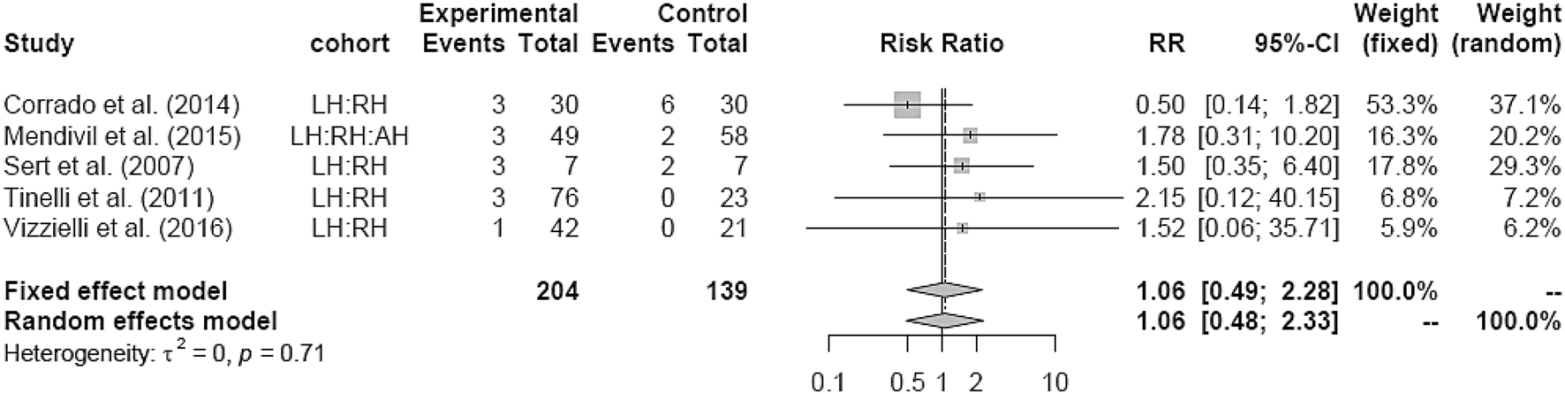
Postoperative morbidity LH vs RH

**Fig. 24:**
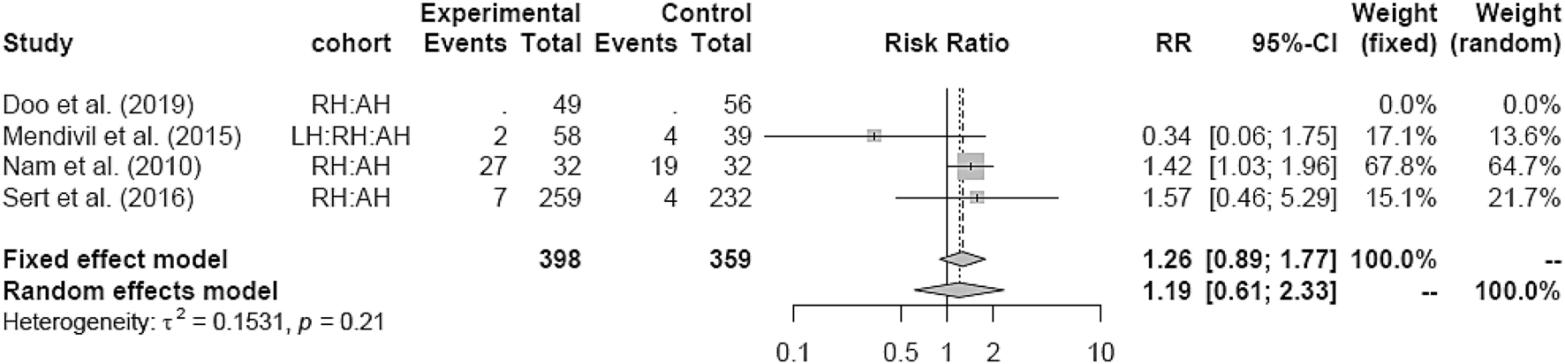
Postoperative morbidity RH vs AH

LH versus RH and RH versus AH showed no significant difference in PM, respectively (Figs. 23, 24).

Subgroup analysis for study design and date of publications in the LH versus RH and RH versus AH groups could not be assessed due to the lack of studies in either one of the groups.

## Discussion

In our meta-analysis of 27 studies, we evaluated the perioperative morbidities of minimally-invasive versus open hysterectomy in early cervical cancer.

LH was associated with lower blood loss and shorter hospital stay as well as equivalent postoperative morbidity compared to AH in the general analysis, even though operation time in the LH group was increased. Intraoperative morbidity was not only found to be lower in the retrospective studies, but also equivalent in the RCT studies and overall analysis. Remarkable in this case is the 95% CI of the overall analysis revealing an upper benchmark only slightly above 1 (RR 0.90 95% CI [0.80; 1.02]), almost also making the IM in general statistically significantly less in the LH group. There was another discordant result comparing only RCT to retrospective studies. The meta-analysis revealed no significant difference of EBL between LH and AH in the RCT group, but a significantly lower blood loss in the LH group of retrospective studies. In the overall analysis, also including retrospective designs, the lower blood loss in the LH group was statistically significant.

In the meta-analyses of Cao et al. [6] and Zhao et al. [7] comparing LH and AH, the results of HS, EBL, IM, PM and OT were concurrent to our analysis. In contrast to our analysis, Zhao et al. included cases of patients receiving neoadjuvant chemotherapy, which could possibly lead to a bias by complicating the situs’ condition.

RH showed lower blood loss and hospital stay, as well as equivalent postoperative morbidity compared to AH. Intraoperative morbidity was lower than in AH, whereas operation time was increased in RH. As well as in LH, the surgeon’s learning curve and the high requirements of laparoscopic techniques could be a reason for the longer operation time than in the more established abdominal approach [18, 19]. Zhang et al. [12] and Park et al. [10] both published meta-analyses comparing RH to LH or AH confirming the safety and effectiveness of RH. All intra- and postoperative endpoints in RH were at least similar to AH, in EBL and HS even in favor of RH, which is concordant to our analysis.

In our analysis, there was no significant difference between LH and RH in any endpoint examined. In several aspects our results are comparable to those of previous meta-analyses but also show differences. Zhou et al. [11] compared RH and LH in their meta-analysis in 2016. Their findings such as less blood loss and shorter hospital stay in the RH group could not be reproduced in our analysis, however it needs to be considered that our search results included only four suitable studies. There is a possible bias in our analysis due to the small number of studies comparing RH to LH.

Our review also has limitations, which mainly involve the heterogeneous and mostly retrospective study designs. Secondly, the smaller patient samples could lead to a bias dependent on the surgeon’s abilities in the field of especially newer operative techniques, such as the robotic hysterectomy. Moreover, we did not distinguish between the different types of Piver classification or additional vaginal approach (LAVH), which could bias the intraoperative complication rate. In addition, several studies combined RH and LH into an overall minimally invasive group, which led to a small number of studies showing results of comparing only RH to either LH or AH. A further limitation is the missing analysis of quality of life in the primary studies. Quality of life after surgical therapy is an important endpoint in need of improvement of evaluation. In the large randomized controlled trial of the LACC study [20], the quality of life were evaluated by standardized questionnaires after a median follow-up of three years. In this analysis, no differences between the abdominal and laparoscopic approaches were seen after 6 weeks and 3 months after surgery. The authors, therefore, conclude to prefer AH over LH due to improved oncologic outcome and equivalent morbidity. Long term results are pending at the time of manuscript editing.

This meta-analysis focuses on patients’ morbidity. The oncologic outcome was analyzed by our research group [17] and showed a dependency of oncologic outcome the surgical approach using various protective measures (no use of uterine manipulator, colpotomy) against tumor spillage. This analysis supports the possible improvement of oncologic safety in minimally-invasive approaches by adapting surgical techniques and was recently supported by the result of a systematic review comparing incidence, mortality and centralization of treatment in early-stage cervical cancer in seven Asian countries. In their review, Hiroko et al. showed that minimally invasive surgery without a uterine manipulator or making a vaginal cuff closure produced similar recurrence rates compared with open surgery (MIS without uterine manipulator vs open-surgery: 10.5% vs 10.1%, and MIS with cuff closure vs open-surgery 7.2% vs 10.1%; all *P* > 0.05) [21]. In addition, the dependence of oncologic outcome on the treatment center was shown in a current cohort analysis [22]. Patients treated in university cancer centers revealed higher survival rates compared to non-university cancer centers, independently from the surgical approach (recurrence-free survival HR 0.49, 95% CI 0.28–0.83; *p* = 0.009 and overall survival HR 0.5, 95% CI 2.06–0.94; *p* = 0.031). However, an analysis in the subgroups applied in our previous study could not be performed in this analysis due to a lack of data in respect to perioperative morbidities.

A meta-analysis of randomized-controlled studies [23] examining the outcome of early-stage endometrial cancer patients treated with either LH or AH showed reduced BL, shorter HS, lower PM in the LH group and no difference in IM. When comparing these results to the findings of our analysis, they differ from ours only in case of PM, which showed no significant difference in our analysis but was significantly lower in LH for endometrial cancer (RR = 0.58, 95% CI 0.37–0.91). Furthermore, no difference in OS and DFS was shown between the LH and AH group of endometrial cancer. Therefore, LH can be considered a safe procedure to treat early-stage endometrial cancer concerning morbidity and oncologic mortality [24].

This meta-analysis and systematic review showed that LH and RH are safe surgical approaches concerning peri- and postoperative morbidities.

## Conclusion

Our meta-analysis showed no significant difference between RH and LH concerning perioperative complications. Operation time was longer in both RH and LH than in AH, but did not lead to a rise in intraoperative complications. Concerning intra- and postoperative morbidity, minimally invasive approaches seem to be superior to open hysterectomy but RCT subgroup analysis did not reveal a difference. Further randomized controlled studies are pending.
